# Infections and cancer: the “fifty shades of immunity” hypothesis

**DOI:** 10.1186/s12885-017-3234-4

**Published:** 2017-04-12

**Authors:** Camille Jacqueline, Aurélie Tasiemski, Gabriele Sorci, Beata Ujvari, Fatima Maachi, Dorothée Missé, François Renaud, Paul Ewald, Frédéric Thomas, Benjamin Roche

**Affiliations:** 1 CREEC, 911 Avenue Agropolis, BP 64501, 34394 Montpellier Cedex 5, France; 2 MIVEGEC, UMR IRD/CNRS/UM 5290, 911 Avenue Agropolis, BP 64501, 34394 Montpellier Cedex 5, France; 3 Unité d’Evolution, Ecologie et Paléontologie (EEP) Université de Lille 1 CNRS UMR 8198, groupe d’Ecoimmunologie des Annélides, 59655 Villeneuve-d’Ascqd’Ascq, France; 4 BiogéoSciences, CNRS UMR 6282, Université de Bourgogne Franche-Comté, 6 Boulevard Gabriel, 21000 Dijon, France; 5 Centre for Integrative Ecology, School of Life and Environmental Sciences, Deakin University, Waurn Ponds, Vic Australia; 6 Laboratoire de Pathologie Oncologie Digestive, Institut Pasteur 1, Place Abou Kacem Ez-Zahraoui- B.P, 120, Casablanca, Morocco; 7 Department of Biology, University of Louisville, Louisville, KY 40292 USA; 8 International Center for Mathematical and Computational Modeling of Complex Systems (UMI IRD/UPMC UMMISCO), 32 Avenue Henri Varagnat, 93143 Bondy Cedex, France

**Keywords:** Immunity, Infection, Cancer, Evolution, Personal history of infection

## Abstract

**Background:**

Since the beginning of the twentieth century, infection has emerged as a fundamental aspect of cancer causation with a growing number of pathogens recognized as oncogenic. Meanwhile, oncolytic viruses have also attracted considerable interest as possible agents of tumor destruction.

**Discussion:**

Lost in the dichotomy between oncogenic and oncolytic agents, the indirect influence of infectious organisms on carcinogenesis has been largely unexplored. We describe the various ways – from functional aspects to evolutionary considerations such as modernity mismatches – by which infectious organisms could interfere with oncogenic processes through immunity. Finally, we discuss how acknowledging these interactions might impact public health approaches and suggest new guidelines for therapeutic and preventive strategies both at individual and population levels.

**Summary:**

Infectious organisms, that are not oncogenic neither oncolytic, may play a significant role in carcinogenesis, suggesting the need to increase our knowledge about immune interactions between infections and cancer.

**Electronic supplementary material:**

The online version of this article (doi:10.1186/s12885-017-3234-4) contains supplementary material, which is available to authorized users.

## Background

Since the beginning of the twentieth century, accumulating evidence shows that some infections may be directly linked to cancer incidence. First, a growing number of pathogens are recognized to be oncogenic, i.e. infection is a prerequisite for maintaining or initiating the growth of cancer cells (Table 1) [1]. Identification of infectious agents that contribute to oncogenesis, i.e. transformation of normal cells into cancer cells, constitutes a priority for cancer prevention mainly because effective preventive measures exist for some of them [2]. Second, oncolytic pathogens, that selectively destroy tumor tissue, have also been studied for more than a century as experimental agents for eliminating cancer cells (Table 2) [3].

**Table 1:** Principal oncogenic agents and their participation to associated cancers

Oncogenic agents	Associated cancer	Contribution	Transmission	Prevention or elimination methods	Carcinogens classification	Ref
Macro-Parasites						[90, 91]
*Schistosoma haematobium*	Bladder cancer	30%	Water	Anti-helminthics		
					Indirect	
*Opisthorchis viverrini* and *Clonorchis sinensis*	Cholangioma liver cancer	15%	Food	Anti-helminthics		
Bacteria						
*Helicobacter pylori*	Stomach cancer	80%	Water, sanitation, food, saliva	Antibiotics, sanitation	Indirect	[92, 93]
Viruses						[92, 94, 95]
Epstein Barr Virus	Burkitt’s lymphoma, nasoparyngeal cancer	10–30%	Saliva	Antivirals for some illnesses		
Hepatitis B and C	Liver cancer	80%	needles, sex	Vaccination (HBV), antivirals, blood screening		
Human T lymphotropic virus	Adult T cell leukaemia	Almost 100%	Sex, needle, milk	No treatment	Direct and indirect	
Human Papillomavirus	Cervical cancer	100%	Sex, saliva	Vaccination, pap smear		
Human Herpesvirus 8	Kaposi sarcoma	Almost 100%	Sex, saliva	No treatment		
Merkel cell polyomavirus	Merkel cell cancer	Almost 100%	Saliva	No treatment		

Today, the World Health Organization acknowledges that at least 20% of cancers have an infectious origin [96]. For transmission section, “needles” includes blood transfusion, contaminated medical syringes and illicit intravenous drug use. A classification of oncogenic organisms has been proposed on the basis of their contribution to carcinogenesis [1]. When infection leads to introduction of viral oncogenes into the host genome, pathogens are considered to be direct carcinogens. These pathogens exploit the host in ways that interfere with mechanisms of cancer prevention (cell cycle arrest, apoptosis, restriction of telomerase and cell adhesion). Infectious organisms that induce immunosuppression, chronic inflammation and/or chromosomal instability, are referred to as indirect carcinogens as they may drive mutations and promote cancer cell proliferation

**Table 2:** Oncolytic agents

More than a century ago, observations revealed that certain natural viral infections (e.g., West Nile virus and mumps virus) were associated with spontaneous cancer remissions [3]. These viruses were subsequently shown to have a natural preference for cancer cells and infection with these oncolytic viruses (OVs) triggers lysis of infected cells as well as activation of anti-tumoral immunity [97]. Advances in molecular biology have also allowed the modification of other viruses to make them specific to neoplastic tissues and/or to combined them with immune reagents to break tumor-induced immune tolerance [98]. For instance, recombinant measles viruses have been used to treat human patients with bone-marrow cancer [36]. Interestingly, this treatment only led to a significant resolution of tumor in two patients who were measles-seronegative. Recently, a genetically engineered virus called T-VEC virus has been approved by the US Food and Drug Administration to treat advanced melanoma [99]. Several studies have also focused on biological anticancer agents based on oncolytic bacteria. In 2014, Roberts and colleagues tested the oncolytic potential of *Clostridium novyi*, a bacterium extremely sensitive to oxygen that permits the specific targeting of cancer cells, in the center of solid tumor, that are in a hypoxic environment [100]. A derivative of the wild-type strain (*C. novyi*-NT) has been engineered to become inoffensive for the host [101] and tested via intratumoral injection against natural canine tumors as well as on advanced leiomyosarcoma in human patients [102]. *C. novyi*-NT destroys cancer cells, but also induces a rapid and robust local antitumoral response. Such experiments pave the way for considering pathogens as new therapeutic opportunities to eradicate neoplastic tissues.	

One alternative and underexplored way to study the relationship between infections and oncogenic events is to investigate the indirect role of infectious organisms^1^ (i.e., viruses, bacteria, fungi, protozoans and metazoans that exploit other organisms, called hosts, to complete their life cycle) that are not considered to be oncogenic or oncolytic in carcinogenesis. These links may result from interactions between immune pathways involved in protection against infectious agents and cancer cells. As the immune system plays a critical role in the control and suppression of malignant cells through immunosurveillance [4], any disequilibrium in immune system homeostasis may enhance or constrain cancer cell proliferation. In addition, infectious organisms could interfere with transmission of oncogenic agents^2^ through partial cross-immunity or immune facilitation, a phenomenon increasingly documented between non-oncogenic pathogens [5, 6]. Therefore, we suggest that oncogenic and oncolytic agents represent the two extremes of a continuum of organisms that play an indirect role during oncogenesis. Since infectious organisms are ubiquitous [7] and co-infections through the course of life remains the norm rather than exception [8], it calls for an urgent need to understand how pathogen communities may prevent or exacerbate carcinogenesis.

## Discussion

### Responses of immune system against proliferation of cancer cells and infections

**Fig. 1:**
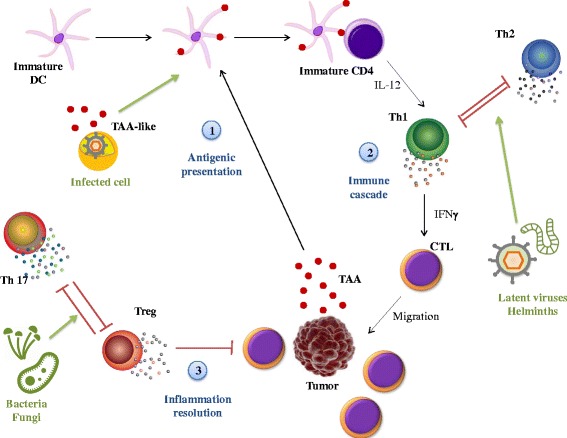
Shared immune responses to infections and cancer cells. The immune system’s action on cancer cells relies on three main steps: antigenic presentation, immune cascades and inflammation resolution. Infected cells can express TAA-like antigen which will activate DC subset. DC will prime Th cells to differentiate into Th1 cells. However, latent viruses and helminths could polarize Th cells toward a Th2 response. Finally, bacterial and fungal infections could disequilibrate inflammation resolution by activating Th17 cells that down-regulate Treg cells. (DC: dendritic cells; TAA: tumor associated antigens; Th: T helper cell; CTL: cytotoxic T cells, Treg: regulatory T cells; IFNγ: interferon γ; IL: interleukin)

While the complex links between the immune system and cancer have been already fully described elsewhere [9–11], it is nevertheless worth pointing out the primary immune mechanisms involved both in infection process and cancerogenesis. For instance, it has been shown that cancer cells are able to evade immune system through numerous mechanisms in advanced stages of tumor [4, 12]. Therefore, we could assume that infections may have a significant role at the beginning of carcinogenesis, i.e. during immunosurveillance. The immune recognition of specific antigens expressed by cancer cells, called tumor-associated antigens (TAA), is a necessary first step to initiate an anti-tumoral response. Receptors expressed on antigen-presenting cells bind and present TAA to T helper (Th) lymphocytes in the lymph nodes. Such Th1-polarized lymphocytes activate cytotoxic T cells and macrophages which in turn destroy cancer cells. In agreement with this, a Th1-polarized response has been mainly recognized to be protective against several cancers [13, 14]. Finally, to avoid auto-immunity and chronic inflammation regulatory T cells (Treg) and other immunosuppressive cells are recruited at the tumor site. Without ignoring that the immune phenotype of an individual results from complex interactions between cellular and humoral effectors, we suggest that mounting an immune response against invading infectious agents could interfere with anti-tumoral protection (Fig. 1).

Indeed, several lines of evidence back up this hypothesis. First, the presence of antibodies against TAA has been observed in cancer-free patients [15], and it has been suggested that some pathogens might have epitopes sharing common features with TAA. In this case, infection with pathogens expressing TAA might play the role of priming the immune response and improve, concomitantly or on a longer time scale, the effectiveness of immunosurveillance. Different infectious agents are known to selectively polarize the immune system towards a Th1 or a Th2 response. Given the reciprocal inhibition between Th1 and Th2 effectors [16], the nature of the infections can have a profound effect on the elimination of cancer cells by immune effectors. This idea is supported by the finding that patients with a Th2-polarized immune response have poor prognosis when suffering from lung, breast, colorectal, pancreatic cancers [17]. Finally, responses against extracellular bacteria and fungi could increase cancer risk through a Th17-mediated inflammation that may also inhibit resolution of inflammation by Fox P3 regulatory T cells (Treg) [18].

As humans are usually exposed to a variety of infectious agents during their lives, it could be expected that the chronology and typology of infections we face, from childhood to old age, might not only shape the functioning of our immune system but also our susceptibility to cancer. Here, we would like to stress that infections are likely to interfere with cancer dynamics at the individual scale depending on i) the personal history of infection (because of the shared immune responses to cancer cells and infections), and ii) the interactions between species within the pathogen communities; but also at the population scale depending on iii) the mismatches^3^ between the environment experienced by our ancestors and our current one (Additional file 1: Figure S1). Finally, we will discuss the possible public health consequences of underestimating such indirect interactions and call for a more integrative view of infectious disease control and cancer prevention strategies.

### Personal history of infection can interfere with destruction of cancer cells

**Fig. 2:**
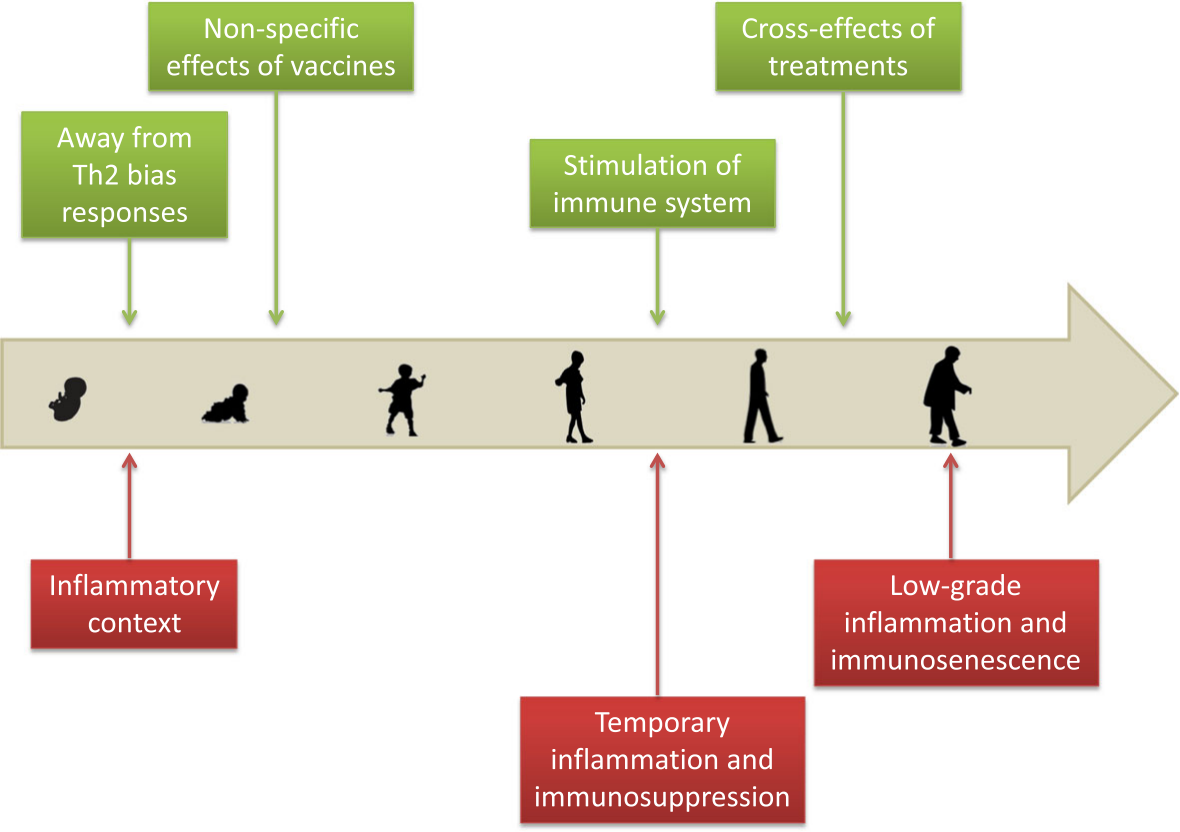
Indirect links between cancer and infections across human life. *Green boxes* and *red boxes* represent beneficial and detrimental links respectively. Childhood diseases and infection events occurring during the life of an individual could reduce cancer risk as they may enhance immune efficiency to eliminate cancer cells. In addition, some vaccines and treatments against infectious diseases have been reported to reduce cancer risk through the activation of anti-tumoral immunity. At the opposite end of the spectrum, infections may create inflammation or immunosuppression episodes that allow cancer cells to proliferate. Finally, chronic exposure to infections could account for age-related immune disorders and the inability to eliminate cancer cells

The community of organisms which have infected an individual during its life represents the personal history of infection. Accumulating evidence suggests that taking into account the past occurrence of infection is important for a better understanding of cancer epidemiology (Fig. 2).

Infections occur as early as the first year of life and may impact the immune system and cancer risk. The increase in antigenic exposure, after birth through viral/bacterial infections, may be essential for newborns to switch from a Th2 biased [19] to a balanced Th1/Th2 immunity as well as to develop immunological memory [20]. Also, childhood diseases may activate specific anti-tumoral responses. For instance, mumps may lead to immune recognition of TAA present on ovarian cancer cells, resulting in an effective immunosurveillance [21]. However, childhood diseases could be associated with inflammation, and the persistence of this inflammatory process in adulthood may increase the risk of mutations in normal cells, giving an example of antagonistic pleiotropy^4^. In fact, individuals that have experienced major childhood illness are twice at risk to develop a cancer [22]. Leukemia is a specific example where childhood infections seem to play an ambiguous role [23]. A protective role of infections was first suggested by observational studies for Acute Lymphoid Leukemia (ALL) [24] and has recently been supported by an epidemiological study for Chronic Lymphoid Leukemia (CLL) [25]. However, another study has reported that the probability of developing ALL increases with the number of infectious diseases encountered in the first year of life [26].

Infection occurring later in life could also have a significant impact on the capacity of the immune system to keep in check cancer cells. Indeed, protection against lung cancer has been observed in humans frequently exposed to cattle in the dairy industry [27]. It has been suggested that protection is provided by endotoxins present in the dust which are known to be potent immune stimulating factors [28]. Furthermore, in a lung-cancer model, mice infected with influenza virus were better able to challenge the tumor [29]. It was suggested that influenza viruses might produce TAA which induces immune memory providing life-long immunosurveillance to cancer cells. The role of respiratory tract infection has also been highlighted by a significant positive association between personal history of pneumonia and CLL risk [30, 31]. Lastly, personal history of infection may also help to explain age-related immunodeficiency, i.e., immunosenescence [32], which is correlated with the reduced capacity to eliminate cancer cells [33]. By increasing exposure to antigens, a longer lifespan may induce chronic low-grade inflammation, contributing to immune disorders, which may, in turn, lead to accumulation of cancer cells in older individuals [34].

Acknowledging the role of personal history of infection in cancer initiation and progression might improve cancer prevention, for instance, through prophylactic cancer vaccination [35]. Consideration of personal infection history could also be useful in treatment strategies as it could alter patient response to therapy. For instance, Russell et al. [36] showed that injection of attenuated measles virus could treat bone-marrow cancer only if patients have never been infected by the virus in the past [36]. This result suggests that immune stimulation may not be high enough when the patient has already been infected by the virus and that the decision to use oncolytic viruses as therapeutic agents has to be made based on the personal history of infection.

Finally, personal history of infection may be related to the personal history of medications and vaccination. Medications that ameliorate symptoms of infection (fever, headache…) may influence carcinogenesis, as is the case for anti-inflammatory drugs. Daily consumption of aspirin, for example, has been recognized to decrease cancer mortality, in part by inhibiting metastasis [37]. Second, medications could be used against specific infectious agents. For instance, the anti-malarial artesunate shows an anti-tumoral activity comparable to other cancer drugs [38]. Also, a range of antibiotics disrupting mitochondrial functions have also been reported to eradicate stem cells of different tumor types [39]. Finally, vaccination against specific infectious agents could be used to prevent cancer. In fact, several studies report protection against melanoma, lymphoma or leukemia after BCG, vaccinia or yellow fever vaccination [40, 41]. These findings might be explained by non-specific effects of vaccines through the shifting of the immune response towards a Th1 profile or through cross reactivity [42]. Vaccines may also contain pathogen antigens with amino-acid sequences that are homologous with those of certain TAA [43]. By this cross reaction effect, vaccination allows eliminating malignant cells as soon as they appear. For instance, a prior immunization with BCG vaccine, which has antigenic similarity with human endogenous retroviruses (HERV-K-MEL), expressed in 95% of malignant melanocytes, has been associated with better survival in patients with melanoma [44].

### Infectious organisms can modify transmission of oncogenic agents

While many pathogens can alter anti-tumoral immunity, some infections can also influence transmission of oncogenic pathogens. Indeed, as with any free organisms, species that form pathogen communities do interact in a synergistic or antagonistic ways [45], with effects on the epidemiology of each species within the community. On non-oncogenic pathogens, it has been shown, for instance, that HIV is responsible for a 37-fold increase in the risk to contract tuberculosis [46] whereas convalescence period induced by measles impacts the dynamics of the epidemic of *Bordetella pertussis* (the causative agent of a whooping cough) [47]. Here, we suggest that this type of interactions has the potential to influence cancer epidemiology by altering the transmission of oncogenic agents.

**Fig. 3:**
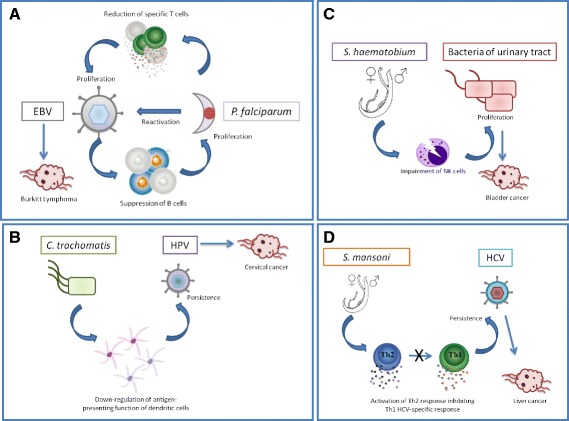
Interactions between infectious agents and oncogenic agents. **a** Reciprocal benefits between Epstein Barr Virus (EBV) and *Plasmodium falciparum*. While EBV suppresses B cells involved in the control of *P. falciparum,* the latter one induces EBV reactivation and decreases EBV-specific T-cells. **b** Helper function of* Chlamydia trachomatis* toward human papillomavirus. *C. trachomatis* products decrease antigenic presentation by dendritic cells allowing the oncogenic agent to persist. **c** Interactions between *Schistosoma haematobium* and bacteria. *S. haematobium* induces the impairment of NKT cells promoting bacterial infections of the urinary tract. **d** Co-infection with Hepatitis C virus (HCV) and *Schistosoma mansoni*. In the presence of HCV, *S. mansoni* has been shown to alter the CD4^+^ T cell proliferative response toward a Th2 profile, preventing the elimination of the virus by specific Th1 response

Endemic Burkitt Lymphoma has been associated with Epstein-Barr Virus infection in infancy and is geographically linked to holoendemic *Plasmodium falciparum* [48]. This association may result from reciprocal benefits for the two species (Fig. 3a). On the one hand, *P. falciparum* antigens can directly induce EBV reactivation and decrease EBV-specific T-cells during malaria infection [49, 50]. On the other hand, EBV in the lytic cycle is associated with suppressed B-cells [51] which play a role in the control of *P. falciparum* [52].

Second, human papillomavirus (HPV) persistence is the major cause of cervical cancer. Epidemiological studies have shown that *Chlamydia trachomatis* infection is also associated with this cancer [53] and increases the risk for persistence of HPV infection [54]. One potential mechanism of this interaction may rely on *C. trachomatis* products which may impact immunity allowing the oncogenic agent to persist. In fact, *Chlamydia* infection induces COX2 protein expression in epithelial cells and promotes PGE2 release [55]. PGE2 has been identified to down-regulate IL-12 production and the antigen-presenting function of dendritic cells [56]. Therefore, *C. trachomatis* infection may increase transmission of HPV by inhibiting cell-mediated immunity but also by creating a pro-inflammatory environment [57] favorable to HPV persistence (Fig. 3b).

Third, *Schistosoma haematobium*, an African trematode that has recently spread into Mediterranean Europe [58], is associated with urinary bladder cancer [59]. Interestingly, several studies have reported a high percentage of bacterial co-infection in the urinary tract [60, 61]. This pattern can be explained by the fact that helminths can induce an impairment of NKT cells promoting bacterial infections [62]. However, bacterial infections of the urinary tract have also been reported to increase the risk of bladder cancer through the production of nitrosamines, which are carcinogenic compounds [63]. Therefore *S. haematobium* could have two facilitating roles in carcinogenesis: a direct role through inflammation-induced DNA damages [64] and an indirect role in immune facilitation (Fig. 3c).

In addition to these well-described examples, evidence of interactions between infectious organisms and oncogenic agents are accumulating for other co-occurrences. For instance, co-infection with Hepatitis C virus (HCV), the causative agent of liver cancer, and *Schistosoma mansoni* has been linked to an increase in viral persistence [65]. In the presence of HCV, *S. mansoni* has been shown to alter the CD4^+^ T cell proliferative response toward a Th2 profile [66], preventing the HCV-specific Th1 response and thus its elimination (Fig. 3d). Specific interactions through the immune system may also occur in the following co-infections: HHV8/*Mansonella perstans* [67] and Merkel cell virus/*Pseudomonas aeruginosa* [68]*,* however, the mechanisms have not been fully identified yet.

Finally, all interactions described above are associated with an increase in persistence/transmission of oncogenic agents while examples of co-infection conferring protection are scarce. We suggest that protection might come from co-infection involving closely related pathogenic species. For example, the immune response against *Helicobacter pylori* (stomach cancer) and *H. bilis* (biliary tract cancer [69]) may be very similar [70], and cross-immunity could result in reciprocal protection. The same mechanism could be applied to co-infections with varicella and HHV8 and/or EBV as they all belong to the *Herpesvirus* genus for which type I interferon plays a central role [71].

### Infectious organisms and cancer susceptibility: an evolutionary perspective

Throughout evolutionary history, humans have been exposed to a great diversity of infectious agents, and the composition of the community has also fluctuated greatly over time [72]. In wealthy countries, mankind has experienced a significant decrease in infectious pressures due to public health strategies, including antibiotics, vaccines, and improved sanitation. The reduced prevalence of infectious diseases has however been paralleled by an increased incidence of many immune disorders, inflammatory diseases, and cancers. One evolutionary hypothesis relies on the mismatch that has rapidly (within a century) occurred between our current infectious environment and the one that our ancestors have been exposed to for thousands of years [73].

#### Infections could drive carcinogenesis by trade-offs^5^ at individual and population scales

The idea that cancer might result from antagonistic pleiotropy (improving early survival and/or reproduction at the expenses of late fitness^6^ (Additional file 1: Fig. S1)) is currently considered to be a viable hypothesis [74]. Nevertheless, very few studies have explored whether traits that help to limit the cost of infection might promote carcinogenesis later in life.

More specifically, resistance against infections could impact pro-oncogenic inflammation. The early immune response to infection relies on acute inflammation [75] which is also accepted as a hallmark of cancer [12]. Despite these oncogenic consequences, the inflammatory response still confers a fitness benefit in environments with high infectious burdens, because it improves the survival prospect at early life. Accordingly, fast-paced species rely more on pro-inflammatory responses whereas slow-paced species tend more toward anti-inflammatory responses [76]. In pathogen-rich environments, pro-inflammatory genes could have been favored, as fitness benefits that arise from early protection against infection would be greater than fitness costs arising later in life, like increased risk of cancer. Pro-inflammatory genes that have been positively selected during human evolutionary history may now be involved in the increased incidence of cancers in modern environments with reduced pathogen loads.

An example of such mismatch comes from the relative vulnerability of African Americans to malignant diseases compared with people of Caucasian origins in the USA [77]. Relocation of Africans from tropical countries, where pro-tumoral inflammation following Th2 activation was beneficial, into North America, and the consequent change in infection risk, may expose them to a higher risk of cancer [78]. Thus, the eradication of some infectious agents – notably those that co-evolved with us – may drive the vulnerability to immune-related disorders, with consequences for cancer susceptibility. While the use of helminths, or at least their immunomodulatory products, has been suggested in treatments of some inflammatory disorders [79], we hypothesize that they could reduce pro-tumoral inflammation, thus pro-tumoral mutation and accumulation of cancer cells. A caveat for such arguments derives from the fact that helminths, like other infectious organisms, evolve characteristics that enhance their own fitness; it is, therefore, naïve to expect that they could have uniformly positive immunological effects on human chronic diseases. If helminths, by their immunoregulatory role, suppress inflammation, they could reduce inflammation-induced oncogenesis. If, however, persistent infection by helminths generates a net increase in inflammation, they could contribute to oncogenesis, an effect that occurs in trematode-associated bladder cancer and cholangiocarcinoma [80].

#### Long-term co-evolution and persistence of oncogenic agents

**Fig. 4:**
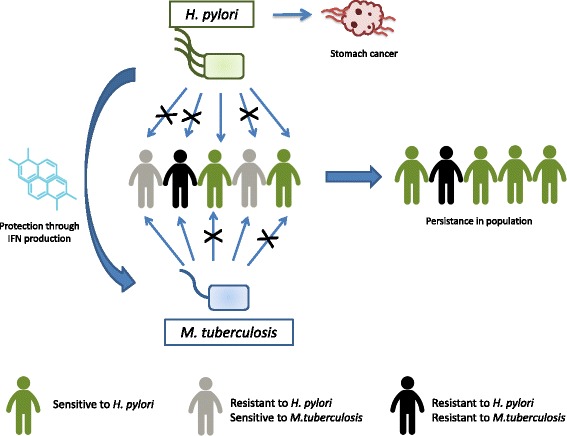
Long term interaction between *Mycobacterium tuberculosis* and *Helicobacter pylori. H. pylori* confers protection against *M. tuberculosis* through an increase in IFN production. In countries with a high prevalence of tuberculosis, infection with *H. pylori* might confer a selective benefit allowing the maintenance of *H. pylori* susceptibility genes

From an evolutionary perspective, interactions between oncogenic agents and non-oncogenic infectious agents are of considerable importance for understanding the dynamics of co-evolution among geographically structured populations evolving under different ecological pressures. When an infectious agent is detrimental to host fitness, selection should favor resistance genes. However, when infections result in net fitness advantages, susceptibility genes should be maintained in the host population. For example, it has recently been suggested that *H. pylori* confers protection against tuberculosis (a lethal disease without appropriate medication) through enhancing IFNγ and Th1-like response to specific tuberculosis antigens [81]. In areas where tuberculosis is highly prevalent, susceptibility to *H. pylori* might have been favored by natural selection (Fig. 4). These conflicting selection pressures could potentially explain the wide distribution of *H. pylori*. Since 1950’s, antibiotics and vaccines have dramatically decreased tuberculosis prevalence in developed countries [82], suggesting that host resistance against *H. pylori* could be selected. Nevertheless, the appearance of resistant strains of *M. tuberculosis* in these populations combined with the increase of HIV transmission could together maintain susceptibility to *H. pylori*. Finally, in countries with low parasite pressure, the persistence of *H. pylori* could also be explained by its protective role against another cancer as it has been reported that elimination of the bacterium comes with an increase in esophageal adenocarcinoma incidence [83].

## Conclusion

In this paper, we put forward several arguments suggesting that the links between infectious organisms and carcinogenesis through the immune system are varied and complex, and cannot be restricted to the study of oncogenic and oncolytic agents. These interactions can operate over the short-term through an altered immunosurveillance (Table 3 summarizes such examples when proximal mechanism has been identified) or via antagonistic/synergistic interactions between oncogenic and non-oncogenic agents, but also on a long-term leading to mismatches. Our arguments stress the need to broaden the view on the interactions between infections and oncogenesis. The interactions, described here to give a glimpse of the overall complexity, also include the microbiota and its possible role on carcinogenesis [84]. Therefore, rather than just studying a simple interaction between one individual and its cancer, we need to explore the intimate connections that could exist with its symbionts sensu *largo* in a given environment.

**Table 3:** Examples of indirect interactions between infectious organisms and cancer through immunity for which the exact mechanism has been identified.TNF (Tumor Necrosis Factor)

Impact on cancer	Infectious organisms	Mechanism implied	Immune compartment	Cancers	References
Exacerbating	Human Immunodeficiency virus	Destruction of CD4 + T cells	CD4+ T cells	Several cancers (including those with infectious origin)	[103–106]
	*Fusobacterium nucleatum* (intra-tumoral bacteria)	Inhibition by contact between bacterial Fap2 protein and immune cell receptor TIGIT	Natural Killer cells	Various tumors	[107]
	*Cytomegalovirus* (infecting cancer cells)	Secretion of immunoregulatory protein (cmvIL-10)	Dentritic cells	Gliomas	[108, 109]
Constraining	*Streptococcus pyogenes*/ *Serratia marcescens*	Secretion of high quantity of TNF	Global immune system	Sarcoma	[110, 111]
	Attenuated Bacillus Calmette-Guérin (BCG)	Local stimulation of CD4+ T cells and Th1 immune response. Diminution of Treg cells.	T cell subsets	Bladder cancer	[112, 113]

From an applied perspective, the stimulation of the immune system is a promising way to target cancer cells without damaging the healthy ones [85]. Most studies have focused on the relationship between immunity and cancer cells elimination based on the understanding of immunological mechanisms underlying the dialog between T-helper cells. Specific antibodies blocking CTLA-4 function enhance T-cell stimulation and promote anti-tumor immunity [86]. T-cell therapies, *e.g.*, those using tumor-infiltrating lymphocytes (TIL) and chimeric antigen receptors (CAR), are promising [87]. Similarly, antibodies have been engineered to block the action of the Th17 cell subset, which secretes interleukin 17, with consistent results in mice where antibody injection was followed by a decrease in the number of tumors [88]. In this paper, we suggest that personal history of infection/medication, including childhood diseases, could modify how the immune system responds to immunotherapy possibly altering its efficiency.

The increase in cancer prevalence has been associated with lifestyle changes, such as an increased caloric intake, urbanization, and sedentary habits [89]. However, infection prophylaxis, improved medicine, and sanitation can also modify the strength of the interactions between infectious agents. In this context, the impact of infectious disease control on cancer epidemiology must be considered. Further work should focus on the potential effect of infectious organisms on cancer incidence and the consequences of infectious disease treatments on cancer risk at different scales. Such a global perspective is indispensable to anticipate the possible consequences of our current public health strategies.

## Additional file

Additional file 1: Figure S1. Antagonistic pleiotropy and mismatch concept. Antagonistic pleiotropy describes a situation where particular genes (e.g. inflammatory genes) have opposite effects on fitness at different ages, such that their effects are beneficial in early life, when natural selection is strong (following infections for instance), but harmful at later ages, when selection weakens. Whereas, mismatches between genotype and environment arise when a phenotype or genotype that were selected in a particular context (e.g. in a high parasitic burden) becomes detrimental in a new environment. (PPT 239 kb)

## Footnotes

1 Infectious organisms: organisms that live obligatorily at the expense of another organism, called the host. The relation is beneficial for the infectious agent but detrimental for the host. This broad definition includes pathogens (virus, bacteria, fungi) and parasites (protozoans, helminthes, ticks among others).

2 Oncogenic agents: Infectious organisms recognized to have a direct and significant contribution to carcinogenesis. At the opposite, we refer to non-oncogenic agents when there is no direct evidence for a contribution in tumoral process.

3 Mismatches between genotype and environment arise when a phenotype or genotype that were selected in a particular context (e.g. in a high parasitic burden) becomes detrimental in a new environment.

4 Antagonistic pleiotropy describes a situation where particular genes (e.g. inflammatory genes) have opposite effects on fitness at different ages, such that their effects are beneficial in early life, when natural selection is strong (following infections for instance), but harmful at later ages, when selection weakens.

5 Trade-off: balance between the cost and the benefit of biological mechanisms regarding the fitness of the organism. It underlies that both aspects compete for a common resource.

6 Fitness: capacity of an individual to produce viable offspring, in other words contribution of an individual to the future generation. Also described as lifetime reproductive success.
