# Supplementary figures and images for: Infections and cancer: the “fifty shades of immunity” hypothesis
Source: BMC Cancer. 2017 Apr 12;17:257. doi: 10.1186/s12885-017-3234-4 (PMC5389015; doi:10.1186/s12885-017-3234-4)

## Slide 1
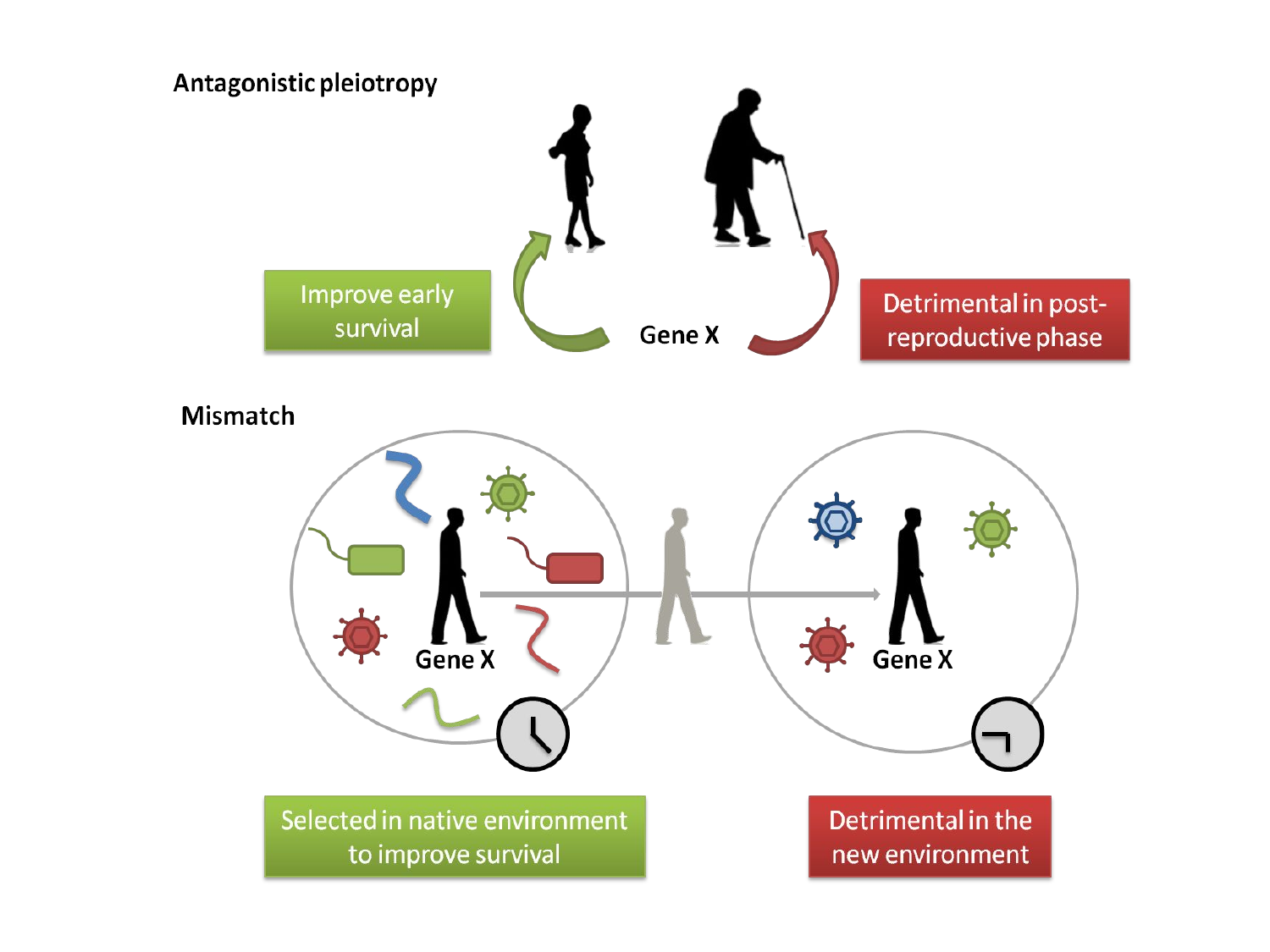

Supplement: Additional file 1: Figure S1. — Antagonistic pleiotropy and mismatch concept. Antagonistic pleiotropy describes a situation where particular genes (e.g. inflammatory genes) have opposite effects on fitness at different ages, such that their effects are beneficial in early life, when natural selection is strong (following infections for instance), but harmful at later ages, when selection weakens. Whereas, mismatches between genotype and environment arise when a phenotype or genotype that were selected in a particular context (e.g. in a high parasitic burden) becomes detrimental in a new environment. (PPT 239 kb) [file 12885_2017_3234_MOESM1_ESM.ppt]
